# What has changed in the diagnostic approach?

**DOI:** 10.1186/2045-7022-1-S1-S52

**Published:** 2011-08-12

**Authors:** Alessandro Fiocchi

**Affiliations:** 1 Macedonio Melloni Hospital, Department of Paediatrics, Milan, Italy

## 

Food allergy in general, and CMA in particolar, are an unique case in which a systematic approach can be applied even to single cases. As the disease involves not only the patient, but the whole family and her social supports, these can be protagonist of the diagnosis itself.

As in any field of medicine, diagnosis starts from the suspicion. In patients reports for milk allergic reactions, an accurate medical history can clarify many aspects of the diagnosis.

If history does not exclude the possibility of a CMA, in particular in delayed manifestations, in primary setting there is the possibility to take a period of tentative avoidance of milk, followed by an open re-introduction. When avoidance coincides with symptom-free periods, an open reintroduction can be useful to identify the offending food (if severe symptoms are anticipated, the procedure should be done under supervision in a medical facility). This elimination - reintroduction phase does not eliminate the necessity of challenge tests, but can give some indication on the possibility of CMA.

We have several methods to evaluate milk sensitization. Basically, they are:

- Skin testing, including immediate skin prick test (SPT), intradermal reactions and atopy patch test (APT)

- The evaluation of serum food -specific IgE using one of the several methods we have at disposition.

Performance, accuracy, and the diagnostic positioning of these have been afforded in the DRACMA Guidelines. Following these analysis, the following considerations can be formulated:

• Challenge is the best for diagnosing CMA

• If not available, challenge is not necessary in case of:

a. high pre-test probability and SPT+ (classify as CMA)

b. high pre-test probability and sIgE+ (classify as CMA)

c. low pre-test probability and SPT- (exclude CMA)

d. low pre-test probability and sIgE- (exclude CMA). • Challenges remain necessary in all cases of uncertainity (medium pre-test probability)

• If challenge is necessary out of a research setting, sensitisation tests may not be necessary.

• Atopy patch test is not useful

• Component-resolved diagnosis may be useful, but further data are necessary

• Molecular diagnosis may be useful, but more data are needed.

